# Formative research to reduce mine worker respirable silica dust exposure: a feasibility study to integrate technology into behavioral interventions

**DOI:** 10.1186/s40814-016-0047-1

**Published:** 2016-02-01

**Authors:** Emily Joy Haas, Dana Willmer, Andrew B. Cecala

**Affiliations:** Office of Mine Safety and Health Research, National Institute for Occupational Safety and Health (NIOSH), Cochrans Mill Road, PO Box 18070, Pittsburgh, PA 15236 USA

**Keywords:** Behavioral intervention, Dust assessment technology, Formative research, Mine health and safety, Risk communication

## Abstract

**Background:**

The use of formative research as a critical component of intervention planning is highly supported in the literature. However, studies that report such processes in practice are minimal. This paper reports on the formative data collection and analysis that informed the development of a multilevel intervention that utilizes mine assessment technology to bridge health communication between workers and management to reduce mine worker overexposure to respirable silica dust.

**Methods:**

Formative research to assess the feasibility and utility of this intervention design included stakeholder meetings and feedback, mine visits and observations, interviews with mine workers, and a focus group with mine management. Data collection took place at several US industrial mineral mine sites and a southeastern regional safety meeting. Interviews inquired about workers’ perceived susceptibility and severity to respirable silica exposure, barriers to preventing overexposure, behaviors that reduce exposure, and perceptions about respirable dust-monitoring technology. A focus group discussed mine stakeholders’ uses of various dust assessment technology.

**Results:**

The data was qualitatively analyzed and coded using a thematic and theoretical analysis. Researchers found recurring themes for both target audiences that informed the need and subsequent development of a mixed-method multilevel intervention to improve communication quantity and quality around dust-control practices.

**Conclusions:**

Results indicate that formative research is critical to: identify and develop an intervention that meets target audience needs; accurately represent the health problem; and develop positive relationships with research partners and stakeholders.

## Background

Mining occurs in a dynamic environment where a variety of internal and external barriers contribute to the challenge of influencing mine workers to engage in health-protective behaviors. Formative data can help identify what internal (i.e., knowledge, attitudes, and perceptions) and external (i.e., environmental) factors should be targeted in a behavioral intervention to modify behaviors [19, 20, 64, 70]. Formative assessments serve many purposes including to: identify, understand, and fulfill needs of a target audience; determine settings for which an intervention should take place; assess changes and trends over a period of time; and inform intervention development at multiple levels [1, 17, 18, 50].

Although an empirical approach to intervention planning that includes formative research is commonly discussed and advocated for in the literature, studies that report such processes in practice are minimal [18, 47, 48, 70]. A literature review conducted by the authors revealed that only a handful of studies discuss formative research for behavioral interventions—often targeting nutrition, physical activity, and safer sex behaviors (e.g., [3, 18, 29, 62, 64, 70]). Further, documentation of using formative methods for the development of occupational health interventions is scarce in the literature. Because occupational health and safety interventions occur in dynamic, varying environments, specific formative research to reveal the feasibility of proposed research is imperative to ensure resonance of the data instruments and applicability of the problem(s) being addressed during interventions with the occupational audience.

The purpose of this paper is to explain both the use and importance of formative research to understand and appropriately address a critical health problem within mining—overexposure to respirable silica dust. There is a high prevalence of silica in materials mined on many operations. Research continues to help lower the levels of respirable crystalline silica in which workers are exposed on a daily basis; however, more work is needed to further preserve mine workers’ health. During this formative research process, researchers explored first a new assessment technology—Helmet-CAM—to identify job tasks and areas of elevated respirable silica dust exposure and to evaluate the potential effectiveness of this assessment technology to reduce respirable silica dust overexposure, and second, ways to integrate Helmet-CAM technology with workers and management to promote behaviors that lead to reduced respirable silica dust exposure. Using guidance outlined in previous research to practice literature (e.g., [9, 22, 50]), National Institute for Occupational Safety and Health (NIOSH) researchers completed several formative research activities including stakeholder meetings, mine visits, individual interviews, and a focus group to understand the health problem, target audience, and feasible solutions. Results were used to develop a behavioral intervention targeting the quantity and quality of health-related communication between mine workers and mine management using dust assessment technology. The contents of the behavioral intervention are not the focus of this manuscript; rather, the formative research efforts that informed the development of the intervention is the focus of this paper. This paper begins with an overview of the health problem that prompted the need for a feasibility study to inform a full-scale intervention.

### Mine worker exposure to respirable silica dust

A current [27] Occupational Safety and Health Objective (OSH-4) is focused on preventing respiratory-related deaths due to respirable silica dust exposures. Because mining is one of the leading industries for occupational exposure to respirable silica dust [26, 28, 35, 58, 63], this occupational health issue is both critical and timely. Mobile mine workers, bagging operators, surface drill operators, and workers for other types of mechanized equipment, including dozers, loaders, and haul trucks have some of the highest exposure rates within the industry [49]. Occupational overexposures to respirable silica dust are associated with the development of silicosis, lung cancer, pulmonary tuberculosis, and airway diseases. Overexposure can also be related to the development of autoimmune disorders, chronic renal disease, and other adverse health effects [53]. Alarmingly, research shows an increasing trend in the number of younger workers, with relatively little mining experience, being diagnosed with silicosis or a related respiratory disease [40].

Fortunately, overexposure to respirable silica dust is preventable. To date, interventions aimed at the prevention of silicosis and other dust-related diseases have focused on the development and implementation of engineering control technologies (for a review of these technologies see [52]). For example, dual-nozzle bag systems depressurize bags after filling is completed, which decreases product blowback, or rooster-tail (89 % reduction in respirable silica dust exposure) and soiled bags (78 % reduction in respirable silica dust exposure). Another highly used technology is an overhead air supply island system (OASIS), also known as a canopy air curtain. Canopies provide an envelope of clean filtered air down over the worker and can reduce silica exposure anywhere from 82 to 98 %. Although these technologies significantly reduce exposure, some workers are still overexposed, prompting the need to integrate a behavioral research component into some technology tools to further mitigate mine workers’ overexposure to respirable silica dust.

#### Helmet-CAM technology

**Fig. 1:**
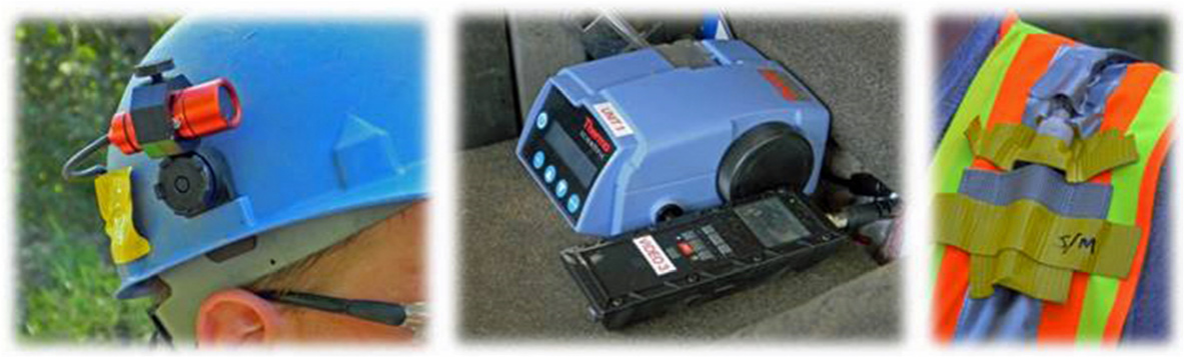
(From *left* to *right*) video camera attached to helmet, dust monitor and video monitor, and safety vest to hold instrumentation

Taking into consideration the mobile nature of several jobs performed by mine workers who often experience elevated respirable silica dust exposures during work tasks, Helmet-CAM technology (Fig. 1) was developed under a cooperative relationship between a mine corporation and NIOSH [6, 7]. The Helmet-CAM system includes a lightweight video camera on a workers’ hardhat and an instantaneous respirable dust monitor on a worker’s belt/backpack. Workers perform their job tasks while video and respirable dust exposure data are collected. Then, video footage and respirable dust data are downloaded to the Enhanced Video Analysis of Dust Exposure (EVADE) software [51]. This software merges the footage and respirable dust data concentration to produce a video that can be played back to help workers and management identify mine work areas and tasks that cause higher respirable silica dust exposures. For more information about the Helmet-CAM and EVADE software, see Cecala et al. [6] and NIOSH [51].

The Helmet-CAM is currently utilized, to some degree, at hundreds of mine sites to identify areas with elevated respirable silica dust. However, worker and management perceptions of the Helmet-CAM, including the potential costs and benefits of using this assessment technology, have not been studied. It is important to understand possible ways with which this new technology can improve worker health to make optimal use of and encourage the adoption of the technology before it is fully integrated into work processes and practices [66]. Although little behavioral research exists around engineering assessment technology, researchers saw an opportunity to help bridge the health communication efforts between workers and mine management using the Helmet-CAM as an intervention tool. However, because researchers had more questions than answers, formative research was necessary to address the feasibility of integrating behavioral research into engineering control mechanisms.

#### Formative research questions

Formative data was needed to narrow the scope of the problem and propose potential solutions to prevent workers’ overexposure to respirable silica dust with this new technology in mind. This formative research explored perceptions from two audiences, mine workers and mine management, to inform ways that management could use Helmet-CAM technology to promote health-related discussions and support mine workers’ participation in healthier work practices to reduce respirable silica dust exposure. The resulting data provided answers to the following questions:

- What are mine workers’ current knowledge and attitudes toward respirable silica dust exposure?
- What behaviors increase/decrease exposure to respirable silica dust?
- What are worker and management perceptions of dust assessment technology?
- How does management currently use, if at all, Helmet-CAM technology to promote health-related discussions with their workers?

The purpose of these questions was to provide researchers with information about the most applicable, feasible solution to help address respirable silica dust exposures on mine sites using Helmet-CAM technology.

### Formative research conceptual framework

Guidelines from applied [9, 22, 50]; and academic literature [25, 43–45, 55, 59] highlight common frameworks and methods used to empirically develop behavioral interventions. The literature indicates that formative research often begins with developing or adapting a conceptual framework that informs the health problem being studied, reveals knowledge gaps, and informs research designs. In the case of mine worker health, researchers used an applied research conceptual framework to initially study the problem.

### Health communication program cycle

**Fig. 2:**
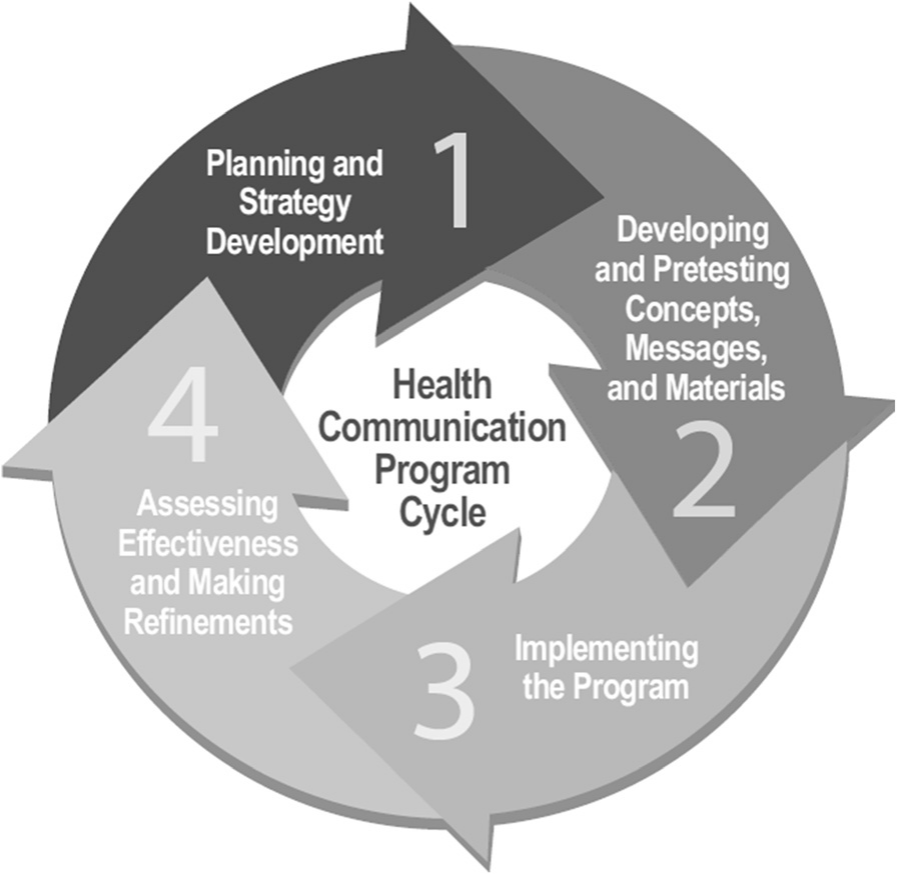
Health communication program cycle ([50], pg. 22)

A practitioner guidebook published by the [50], *Making Health Communication Programs Work*, also known as “The Pink Book,” provides a four-step cycle to inform health communication program development (Fig. 2).

Literature within occupational health and public health marketing offers similar program development processes. For instance, CDCynergy [9] is a CD-ROM-based tool that provides a step-by-step process for developing and implementing a health program. NIOSH advocates a similar cyclical process, but distinguishes partnership development from planning and strategy development [22]. A majority of published literature focuses on phases 3 and 4, implementation and evaluation. This paper details the initial phases of these cycles—strategy development and planning, and pre-testing concepts and materials. In other words, phases 1 and 2 of these applied framework models specifically encompass formative or pilot research. However, the documentation of these phases often is absent in final results of such studies.

## Methods

**Fig. 3:**
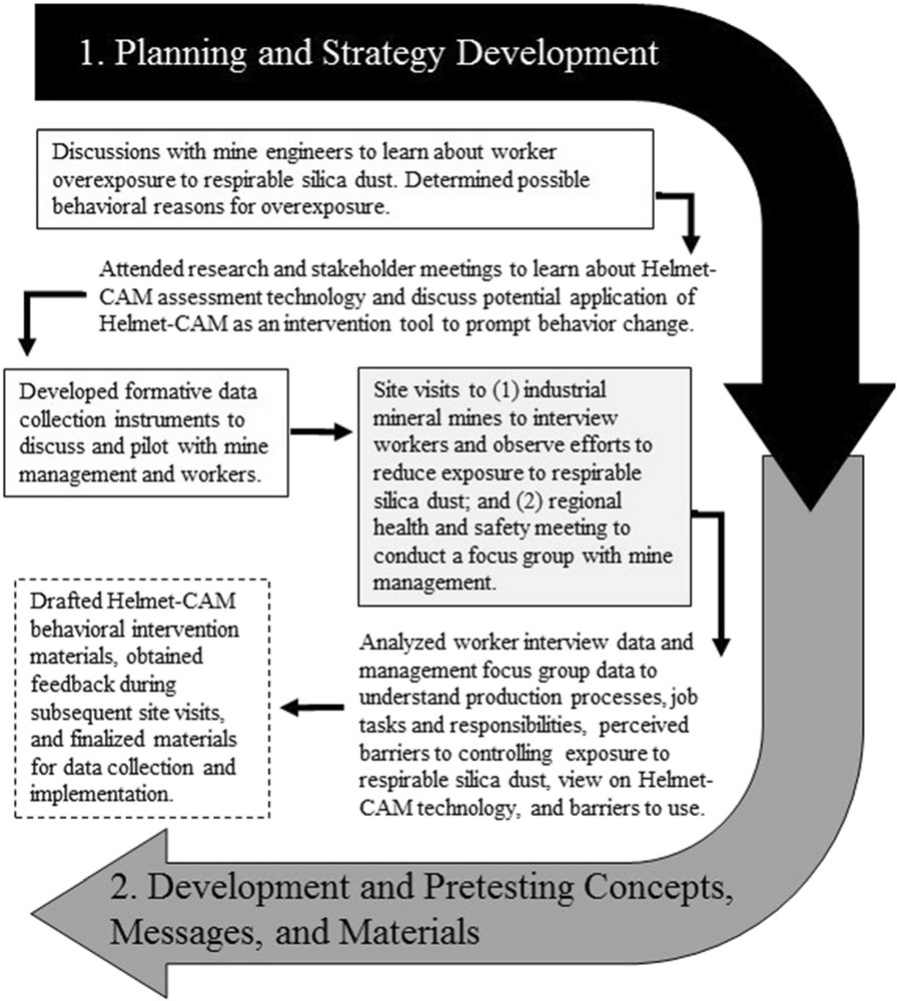
Formative research process

Because stakeholder meetings are informative and help foster important partnerships [44, 50, 64], researchers participated in meetings with mining engineers and stakeholders to better understand respirable silica dust exposure and Helmet-CAM technology before collecting data. Subsequently, qualitative methods including interviews, a focus group, and observations were used to more clearly identify and define the health problem and target audience [4]. Data collection was ongoing from January through October, 2014. Figure 3 highlights the efforts completed during the formative research process.

### Phase 1: planning strategy and development

Planning strategy and development included identifying the health problem, understanding the target audience, crafting a solution strategy, and drafting initial data collection tools to help minimize negative reactions from the target audience during implementation [9, 34, 50]. Planning strategy and development activities occurred during multiple partner/stakeholder meetings where previous research efforts were discussed.

#### Partner and stakeholder meetings

Soliciting expert knowledge from key individuals helped determine the full scope of mine worker overexposure to respirable silica dust [50]. First, regular meetings occurred with mining engineers who study respirable silica dust exposure and engineering technologies. These engineers also expressed the need to integrate a behavioral component into current respirable silica dust exposure research. Engineers worked with researchers to understand the full scope of the problem and identify potential solutions, with Helmet-CAM technology in mind. For example, mining engineers informed the social science researchers that bagging operators are continually overexposed to respirable silica dust, despite consistent application of viable control technologies. Researchers also learned about the history and previous uses of the Helmet-CAM, including how the assessment technology was received and used by the mining industry during initial field tests [51]. This knowledge allowed researchers to accurately communicate about respirable silica dust and the Helmet-CAM technology during subsequent stakeholder meetings and mine visits. Meetings were held that involved industrial hygienists, engineers, and mine health and safety personnel who expressed interest in using the Helmet-CAM as an assessment tool. During stakeholder meetings, researchers had the opportunity to explore perceptions of Helmet-CAM technology and its utility as a behavioral intervention device. Support garnered during these meetings resulted in further research links with mining engineers and invitations from mines that provided researchers with opportunities to observe and further characterize the utility of Helmet-CAM technology. Information learned during these meetings helped researchers to draft the targeted questions for formative data collection in the field.

### Phase 2: developing concepts, methods, and materials to pre-test during data collection

The next phase involved drafting instruments to engage mine workers and mine management to help researchers comprehend and reduce respirable silica dust overexposures [50]. Researchers drafted qualitative data collection instruments to investigate worker and management perspectives about respirable silica dust exposure and assessment issues. In addition, a draft intervention framework was developed so researchers could request initial feedback from mine management. All of these formative materials were approved by the NIOSH Institutional Review Board (IRB) before mine visits. Data was collected during mine visits and during a regional safety meeting off-site. Each individual provided informed consent before participation.

#### Interviews with mine workers

Although all workers face the potential of overexposure to respirable silica dust, bagging operators (i.e., baggers) have among the highest overexposure rates in mining (26 % under the current regulation) [49]. Bagging operators are involved with the process of filling 50- to 100-lb bags with a respective mined product and then stacking the bags on pallets, to be lifted, using a forklift into a shipping vehicle [8]. Typically, bagging operators work at a filling station that is equipped with a fill nozzle from which the operator hangs an empty bag and pushes the start button to fill the bag with product. As each bag is filled, either an automated process mechanically ejects the bag onto a conveyor belt or the bag machine operator manually removes the bag and places it onto a conveyor [8]. During this process, these operators are exposed to multiple dust sources [65]. Therefore, 12 baggers at silica sand operations were recruited and consented to participate in an interview (three pilot interviews and nine semi-structured interviews). Pilot interviews occurred first to pre-test the questions and ensure that the researchers were obtaining useful information and/or if question revisions were needed [39]. Although opinions vary, anywhere from 6–12 individual participants are common for interview data collection procedures [32, 38, 41].

### Recruitment and participants

Mine health and safety managers were contacted via phone or e-mail and were informed about the purpose of the information collection. After granting mine site visits, managers offered their bagging operators the option to participate. Interviews were conducted at two industrial mineral plant operations in July and August, 2014. One operation in West Virginia had an automated bagging system in which two workers were assigned to monitor and work near the bagging system. The other plant, located in New Jersey, did not have a fully automated system and required five workers to rotate tasks throughout the day to complete the bagging process from loading to palletizing. Participants were male and their mining experience ranged from 4 to 37 years. Each interview required approximately 20–40 min to complete. Although experience of baggers varied, similarities across their knowledge, attitudes, and behaviors emerged, reaching saturation of data by the end of recruitment interviews at both mines [13].

### Instrumentation

An interview guide was drafted to probe workers’ risk perception and health behaviors. Health behavior theories including the protection motivation theory [56], the health belief model [30], and the precaution adoption process model [67] were referenced to devise questions about workers’ health-protective intentions and behaviors. For example, baggers were asked to discuss perceived barriers to specific behaviors that minimize exposure to respirable silica dust. Participants also discussed hazards that they encounter during work tasks, the work tasks that expose them to the most respirable silica dust, if and why dust exposure is something that they are concerned about, and what they personally do to reduce their exposure to respirable silica dust on their respective mine sites.

#### Focus group with mine management

Mine health and safety managers are responsible for acclimating workers to new technologies. Therefore, it was necessary to investigate how managers were currently using Helmet-CAM technology and how it was viewed. A focus group with nine individuals was conducted with members of mine management. Generally, focus groups consist of 8–12 participants [2] but can have as few as 6 [5]. Although researchers did not achieve an optimal number of focus groups, which is generally three [37], the formative aspect of the research deemed the focus group appropriate to obtain the desired information.

### Recruitment and participants

The vice president of health and safety for a major mine corporation invited researchers to an annual regional safety meeting to learn more about their current health and safety goals. The vice president also enlisted the organizer of the regional meeting, a health and safety manager in the area, to recruit individual attendees who were eligible for participation (i.e., those with knowledge of and experience using Helmet-CAM technology). The focus group occurred at a large mine corporation’s southeast regional health and safety meeting in North Carolina in October 2014. Female and male safety managers were present, all with a range of experiences in their current management position. Multiple mine sites were represented during the 60-min discussion.

### Instrumentation

Researchers developed a focus group protocol to engage site-level health and safety management. Questions focused on ways management communicates with employees about health and safety. As the discussion continued, the co-facilitators asked management about personal uses and benefits of the Helmet-CAM technology at their mine site and how it can be used to communicate with workers in a way that illustrates support for healthier work practices. In addition, managers were asked for feedback about ways to improve the EVADE software that merges the instantaneous dust monitor data and video in the Helmet-CAM technology. Information was captured via note-taking during the discussion.

#### Mine visits and feedback

Observations occurred at three mines during 3-day visits to each site to complement the qualitative data and help researchers understand what and why certain solutions to respirable silica dust overexposure may be desired. More specifically, observations provided additional context about employee behaviors and interactions, which were referenced during the interview, and focus group analyses and intervention development [3, 42].

During the observations, researchers asked workers and management questions about their mine processes and risks. Some workers wore the Helmet-CAM technology because management was interested in integrating the assessment technology at their site but not sure how the response would be on site. At one site, researchers were able to observe if and how wearing the Helmet-CAM affected workers’ job tasks and personal behaviors, and to hear workers’ feedback to their management about wearing the device. In addition, the proposed intervention design, focused on targeting the quantity and quality of health-related communication using Helmet-CAM technology, was discussed with management at all three sites. Feedback about terminology and utility of the instruments was obtained to help tailor tools to be more resonant with the culture at each mine. For example, managers at one site indicated that instead of using “manager” in the survey, using “coach” would be more applicable to how their organization is structured. Another comment spoke of the number of site visits feasible to include in the intervention.

### Data analysis

Qualitative data was analyzed independently by the target group (workers and management) and then considered together to inform aspects of the intervention design. Researchers completed the thematic analysis of the mine worker interview data first. Written notes were examined to identify trends in baggers’ perceptions related to respirable silica dust exposure, consequences to overexposure, and work practices to reduce overexposure. Researchers sorted the data into categories based on general risk perception inquiries discussed within health behavior theories and the research questions inherent in the study design—with a specific focus on knowledge, attitude, and behavior [15, 69]. After considering perceptions of workers, focus group information was referenced to provide a more accurate description of mine site exposure levels and general work practices that effectively reduce exposure to respirable silica dust. Focus group notes were analyzed to help understand the actual and potential impact of Helmet-CAM technology at mines.

Open and focused coding techniques were used within a grounded theory (GT) approach to make iterative inferences about what the data might mean [10, 57]. GT is a research tool that focuses on constant comparison of data to allow social patterns and structures of interest to emerge [10]. GT was chosen as an appropriate methodological approach to systematically gather and analyze field data. Because GT involves moving in and out of data collection and analyses, which occurred throughout the duration of the various formative research efforts, it was possible to develop initial answers to research questions and then add to those responses over time through open, axial, and eventually theory-driven coding [61].

Theory-driven coding was used in tandem with GT to link any emerging themes to theoretical constructs that informed interview and focus group questions [24]. During theory-driven coding, researchers integrated participant examples into the already-identified themes to consider how the individual narratives might relate to broader, more inclusive issues within the problems and solutions of respirable silica dust exposure. Emergent themes were organized into files. These files also contained memos, or notes, based on the informal interactions researchers witnessed between workers and management [42]. These notes helped researchers centralize commonalities between target audiences and characterize reasons for workers’ current knowledge, attitudes, and behaviors related to respirable silica dust exposure.

## Results

This exploratory research provided valuable information about workers’ respirable silica dust exposure and the barriers to and benefits of using Helmet-CAM technology in the future to educate about and reduce exposures. Typically, knowledge, attitude, and behaviors are represented within theoretical frameworks to inform the development, implementation, and evaluation of behavioral interventions [15, 22, 48, 50]. Therefore, data was analyzed, and results are organized around these three areas—knowledge, attitudes, and behavior—to present viable options and information for future intervention development and implementation at mines.

### Mine worker knowledge of respirable silica dust sources and exposure

Baggers who participated unanimously indicated that their primary health risk on the job is exposure to respirable silica dust. Participants were knowledgeable of specific job tasks that may produce more respirable silica dust and overwhelmingly indicated that these tasks are their least favorite. For instance, one bagger said, *“I hate dry sweeping because of all the dust it kicks up.”* Another bagger said, *“I like driving [the fork lift] the best, it goes quick and you have less dust risk too.”* Job tasks with higher exposure risk include loading and sealing bags and cleaning up broken bags. When using Helmet-CAM technology in future research, these tasks would be appropriate to observe and assess.

Participants acknowledged that respirable silica dust constantly circulates in the air and is particularly noticeable when rays of sunlight shine into the facility. One bagger said, “*It’s crazy how much dust is actually in the area. So if I see that I usually put on the respirator even if it’s not required.”* Baggers said that seeing dust is a good reminder to wear their respirator. This situation provides anecdotal evidence that appropriate messaging and immediate feedback regarding working conditions may prompt workers to engage in a health behavior (i.e., wearing a respirator).

A few of the baggers were already aware of Helmet-CAM technology because their managers had previously asked them to wear the device for field testing purposes. These individuals reported positive experiences while wearing it during a work task cycle and appreciated the quick feedback about their high exposure areas. The individuals who were not familiar with the assessment technology expressed interest in learning how the Helmet-CAM works and perhaps wearing it in the future. These attitudes suggest Helmet-CAM technology may be a viable intervention tool for this audience and setting.

### Mine worker attitudes toward personal health

Attention to respirable silica dust exposure and silicosis was more prominent at the onset of participants’ employment, but as they became more comfortable with their job tasks, this attention diminished. For instance, one bagger said

*Dust exposure scared me more when I first started here eight years ago. I don’t worry as much now. I don’t know; maybe I’ve just gotten complacent. But really I’m 31 so I don’t have to worry about dust exposure for another 20 or 30 years. So I think about it but not that much.*

Responses from other participants also suggested that length of tenure might play an important role in levels of perceived risk. Participants with a moderate length of experience (i.e., 5–15 years) tended to say that risks (for overexposure) are lower due to required respirator-use in certain areas and the plant’s compliance with respirable silica dust regulations. One bagger said, *“I know I’m at certain risks for cancer and things like that but not for a while.”* Related to screening for silicosis, one bagger said, *“I really don’t think much about my health because every two years we get a physical. I have no signs of any problems so I’m fine.”*

In sharp contrast, participants who were nearing retirement expressed increased awareness of and vulnerability to dust-related diseases. A bagger with 36 years on the job stated, *“I think about silicosis all the time, every day.”* These responses indicate that communication with mine workers about their health may need more attention during middle stages of workers’ careers, when complacent attitudes and low perceived susceptibility may influence more lax personal dust-control practices. Targeting this segment of workers with a behavioral intervention may be appropriate.

### Mine worker protective health behaviors

Participants were asked about personal protective behaviors that they engaged in to reduce respirable silica dust exposure. All participants reported wearing their respirator at some point even if the respirator was not required in a particular area—because they were personally concerned about exposure levels. For instance, when dealing with finer grades of silica sand products which are “dustier,” several participants noted that they elect to wear their respirator more often. Other behaviors mentioned included closing doors when they see dust, changing where they position themselves in proximity to an area with higher respirable silica dust sources, requesting that water trucks circle the area more often, and adjusting curtains to improve ventilation. Helmet-CAM technology could be used to assess the usefulness of these practices.

Despite these protective health behaviors, participants also felt that there is not much else that they can do to prevent overexposure to respirable silica dust. To illustrate, baggers generally said, *“You can’t add much or do much to protect yourself besides wear your respirator,”* or “*There’s not a lot left that you can do in bagging. I think we have done everything possible to control dust.”* The fact that baggers felt a sense of impossibility when it came to further lowering their respirable silica dust exposures is a topic worth addressing within mines. Perhaps using Helmet-CAM technology to show workers that their behaviors can make a difference in personal risk would be a step toward worker engagement and empowerment.

### Mine management knowledge of Helmet-CAM technology

The purpose of talking with mine management was to ascertain current levels of respirable silica dust and the attitudes toward and uses of Helmet-CAM technology. It was important to anticipate negative feedback from potential participating mines and how researchers could minimize concerns. Because a requirement for participation in the focus group was familiarity with the Helmet-CAM system, all participants were knowledgeable about the technology. Most of the managers had been using the Helmet-CAM system to assess workers’ exposures on a semi-annual or as-needed basis for at least a year. Because this technology is not widely utilized in the mining industry, this feedback from subject matter experts was highly valued.

Although the baggers interviewed did not express concerns about wearing the Helmet-CAM system, management who participated in the focus group indicated some of their workers express concerns about “being watched” while performing their duties. They also reported that some employees complain that the device is too heavy and a “little annoying” to wear. This information, contradictory to individual worker feedback, needs to be considered during future interactions with mine workers who may participate in an intervention and wear the Helmet-CAM system during a work task.

### Mine management attitudes toward Helmet-CAM integration

Managers discussed advantages of Helmet-CAM technology, but eagerly shared their frustrations with it as well. Many found it to be somewhat burdensome as part of their job responsibility based on functional issues with the software. For instance, managers indicated they would not want to keep using the technology if the software and general system do not improve, even though it has several benefits. They expressed “growing pains” when starting to use the technology, including saving and retrieving files in the correct place. Based on these concerns, researchers adjusted the project timeline to postpone any interventions with Helmet-CAM technology until the updated version of the EVADE 2.0 software became available during summer, 2015, for mine leadership to download and use for their personal assessments. The updated version is more user-friendly and should help to alleviate most of the concerns expressed by participants which we thought would be helpful for garnering mine site participation in the future.

### Mine management action and environmental changes based on Helmet-CAM

Managers had general knowledge of and experience using Helmet-CAM technology as an area sampling tool to find sources of major respirable silica dust that they were not able to identify on their own (e.g., environmental issues such as leaks around their facility). In general, they reported that it is a good tool for both procedural and environmental issues. Most described a process of using the Helmet-CAM to identify an issue, correct the issue, and then evaluate and confirm the correction in a post-test. Participants also indicated that Helmet-CAM technology has been effective in changing some work behaviors and even discovering new, best work practices. For example, they found that when changing a screen, exposures to respirable silica dust are significantly lower when the screen decking and clips are vacuumed first. Therefore, it is plausible that this technology may help inform and influence healthier work behaviors, if strategically discussed with workers.

## Discussion

### Implications for practice

Aligned with the benefits of formative research referenced earlier, ineffective health programs are often cited for too little effort to understand target audiences, develop purposeful data collection materials, and identify leverage points to reach the target audiences [44–46]. This current research process confirmed the importance of formative data to help recognize worker and management perspectives related to respirable silica dust exposure and consider viable, reliable solutions to address the problem in future research. As a result of this formative process, researchers had ample data to inform the development of a 6-week repeated measures behavioral intervention targeting both mine workers and mine management (refer to Federal Register 79 [14], pg. 68447 for a description of the longitudinal intervention design). Aspects of this intervention that were particularly informed by this formative research are described below.

### Identifying a practical solution

It is widely acknowledged that desired work priorities can and should be expressed, monitored, and consequences should be delivered during everyday communication between managers and workers to encourage healthy work practices [33, 72, 73]. Therefore, it was determined that if an intervention could improve the quantity and quality of communication between management and workers, the health behaviors of workers may improve as well. This type of data from workers can provide feedback to management about ways to improve their health communication strategies with employees. For example, baggers who were interviewed indicated that communication from management does influence health behavior (e.g., wearing a respirator to avoid a negative interaction).

While wearing a respirator is a visible work practice, results indicated that busy agendas may preclude management from knowing where, when, and why their employees struggle to reduce exposures around the mine. Focus group participants indicated that Helmet-CAM technology could help solve some of these visibility issues by providing a unique vantage point for management to see the areas where workers face higher exposures and why. As an example, focus group participants discussed high exposures inside grinding mills when workers have to repair or replace liners and suggested the information provided by the Helmet-CAM system as useful for management.

Through interacting with employees and monitoring exposure rates, management may better identify the types of interactions and messages that work best with their employees. This information can be used to develop best practices and tailor communication with workers about health and safety.

#### Tailoring the solution

Tailored communication that resonates with the target audience is critical for program effectiveness [9, 34, 44, 45]. Tailoring enhances information relevance and has been shown to produce greater changes in health behaviors [31, 36, 60]. Therefore, the intervention is designed to provide mine management the ability to tailor their communication with each mine worker. After management reviews worker experiences from the Helmet-CAM technology with an employee, they can communicate about specific areas in the mine and mutually agree what work and/or environmental practices can be modified.

Through these tailored conversations, management’s goal is to enable workers to strengthen skills, use resources, increase efficacy, and to gain better control over their health outcome [71]. This process of meeting workers where they are and helping them solve exposure problems helps reinforce self-efficacy and can ultimately lead to sustained behaviors [24].

### Collaborating to solve a problem

Effective intervention development requires a great deal of collaboration and communication. Researcher involvement with mining engineers and stakeholders was ongoing. Results demonstrated that the formative research process helped establish collaboration and acceptance of the research goals. In addition, integrating this step early helped demonstrate concern and interest in the problem to mining stakeholders, eventually building trust and acceptance among the target group and potential partners [64]. These results further support continued collaboration to address health problems within a specific community [25].

#### Involving social scientists in technology integration

New technology is increasingly common in the mining industry. Although not common in practice, research supports human factors or social science involvement during early planning stages through the design and utilization of engineering technology solutions [11, 12, 16]. In the current study, collaboration between social scientists and engineers facilitated a greater focus on aspects of human/technology integration. For example, researchers were able to question mine workers and management and obtain observable feedback about the Helmet-CAM system in action, to relay to mining and technology engineers, before wide dissemination. Because the occupational workforce is more likely to accept a new technology if given a voice in its implementation [16], these efforts may help prevent resistance and/or other negative reactions in the future.

Likewise, analysis of data from the formative research phases revealed the importance of including mine management in the technology integration process. Generally, middle management has been a significant obstacle to the adoption of advanced technology [16]. By giving these middle managers a specific role in integrating the technology, they have more control over how, when, and what they communicate with their workforce about healthier work practices. Collaborative relationships fostered during the current formative research elucidated the types of communication that needs to occur at mines about health behaviors. Without working together, the human (social science) and technology (engineering) integration may have been overlooked.

#### Garnering support for future participation

Engaging stakeholders early in the planning process, to obtain expert feedback, allowed researchers to present the problem and brainstorm solutions independent of recruiting efforts. Members of management who participated expressed that the proposed intervention design is feasible and desirable within the industry. For example, when discussing this design with a health and safety official, that official mentioned that a common theme/concern that came out of brainstorming sessions during a workshop is the communication gap between management and hourly workers (personal communication, 19 October 2014). Although not a primary goal, these engagement efforts safeguarded effective implementation in the future [23]. Because communication and Helmet-CAM technology are both desirable areas to improve within the industry, stakeholders who provided expert advice also offered their mine sites for participation in hopes of improving these areas within their own organization.

### Limitations

This research should be considered in light of its limitations. First, the mine worker sample of baggers should be expanded to include other job roles and tasks in future interventions. However, to assess the feasibility of this work, the researchers felt it was important to focus more specifically on one audience to better understand their perceived barriers to elevated respirable silica dust exposure on the job. However, the researchers aimed to include mines of diverse production and size to obtain a broader sample in this regard. Collecting additional samples would arguably have provided greater depth, which is desirable. Researchers found, however, being present at each mine for multiple days, conducting observations and informal questioning, provided sufficient additional context for analyzing the interviews and focus group for the purposes of this phase. Similarly, showing the intervention materials to a greater number of mine sites to obtain information would have been preferable. However, due to the tailored design of these interventions (i.e., management has the ability to communicate individual messages to each worker), the intervention may appear different at each mine. In addition, although the overall sample was not large, both target audiences were consistently engaged, using guidance from health behavior theories. Because researchers observed consistent themes across the knowledge, attitudes, and behavior spectrum, the data was deemed appropriate to better understand the health problem, target audiences, and inform intervention development.

## Conclusions

Despite the limitations of this research, the aforementioned process provides not only a more reliable approach to intervention development by adhering to best practices in the literature, but also illustrates how to begin solving the persistent problem of translating research findings to practice. First, completing and documenting the initial phases of intervention development through planning and pre-testing fills a gap in behavioral intervention pilot research [47, 48]. Utilizing constructs from multiple theories to understand the audience also helps inform the development of tailored interventions and is considered a common research practice [54]. Because health behavior theories were considered during formative instrument development, researchers received feedback from both audiences that was grounded in theory and that could be applied to future field-based research. Also, although these interventions are not yet evaluated, these initial needs assessments provide a base to gauge applied effectiveness in the future [22, 50]. Engaging in a post-intervention effort that contains similar data collection activities applied during these formative research phases may be useful to understand areas of the intervention that worked better than others.

Second, it is rare for research designs to be implemented in applied settings [21], and practitioners argue that they have insufficient evidence to make and carry out recommendations (e.g., [9, 68]). While these same problems exist within mining, if the current intervention design is initially overseen by researchers, in tandem with mine management, research efforts can be more transparent, and the industry may be more likely to continue applying solutions. Therefore, upon implementation and evaluation of these interventions, specific recommendations for using Helmet-CAM technology as an educational and communication tool to improve health behaviors can be made to industry. It is hoped that this formative process and subsequent intervention design informs applied research practices in several areas of study that can be executed on behalf of leadership as a part of their own organizational health and safety research strategies.
